# The clinical efficacy of Shengyu decoction in the treatment of anemia after PFNA for elderly intertrochanteric fracture: A protocol of prospective, randomized, controlled trial

**DOI:** 10.1097/MD.0000000000028812

**Published:** 2022-02-11

**Authors:** Wei Lu, Wenhao Zhu, Yu Xiao, Hao Hu, Yunlu Zhang, Yanqi Feng, Hongbo Wan, Zhaoxiang Fan, Xuequn Wu

**Affiliations:** Longhua Hospital, Shanghai University of Traditional Chinese Medicine, Shanghai, China.

**Keywords:** anemia, intertrochanteric fracture, proximal femoral nail antirotation, protocol, Shengyu decoction

## Abstract

**Background:**

Femoral intertrochanteric fractures (ITF) occur frequently in the elderly, accounting for 45% of all hip fractures. Postoperative anemia after fracture tends to cause ischemia, hypoxia in cells, tissues and organs, increasing the rate of blood transfusion, risk of infection, disability and mortality. Meanwhile, traditional Chinese medicine is widely used in the treatment of anemia for activating blood circulation and removing blood stasis.

**Methods:**

This study is a prospective, outcome assessor-, and data analyst-blinded randomized controlled clinical trial. The objective of this proposed study was to investigate whether Shengyu Decoction could improve the symptoms of anemia after proximal femoral nail antirotation in elderly ITF patients. After qualifying for screening, patients will be randomized into 2 groups with an allocation ratio of 1:1. Hemoglobin concentration, HBL, and HHS score are outcome measurements. The other outcomes also included time to get out of bed, discharge to home, 30-day readmission rates, and mortality.

**Discussion:**

ITF is commonly occurring in senior citizens, and those who are senior in age generally suffer 1 or more basic diseases, whose nutritional status is already poor. Trauma and surgical stimulation not only aggravate the existing disease or induce corresponding cardiovascular complications, but also worsen the nutritional status, which can easily cause postoperative anemia in patients. Because of the limited clinical modalities available for the treatment of postoperative anemia after fracture surgery, and most of them have various side effects that are not easily tolerated by the elderly. Therefore, from a traditional Chinese medicine perspective, we proposed a protocol using mild Chinese herbal decoction to treat postoperative anemia in ITF.

Registration number: OSF Registration number: DOI 10.17605/OSF.IO/JQ6ZF.

## 1 Introduction

Femoral intertrochanteric fractures (ITF) occur frequently in the elderly, accounting for 45% of all hip fractures. With the acceleration of the aging population, the incidence of femoral intertrochanteric fractures is also increasing.^[1,2]^ At the same time, complications caused by long-term bed rest in nonsurgical patients often lead to high disability and high mortality. Survivors are prone to various sequelae, making this disease an important public health problem. In addition, the high cost of treatment makes it difficult. Both national health and social economy have a serious impact.^[3]^

Surgery is currently recognized as the main treatment method, which can reduce pain, promote joint functional recovery, avoid many complications caused by long-term bed rest, and improve the quality of life of patients.^[4]^ Among them, the proximal femoral nail antirotation (PFNA) has the advantages of easy operation, less trauma, and stable fixation. Its characteristic is that it maintains the firm fixation and biomechanical stability of AO. The essence of BO and minimally invasive surgery is suitable for various types of femoral intertrochanteric fractures, and is currently one of the main clinical surgical methods.^[5,6]^ However, with the popularization and application of PFNA in the treatment of ITF, more and more scholars have found that although the amount of bleeding during the operation of PFNA is small, some patients show different degrees of anemia after the operation. Li analyzed the perioperative blood loss in 123 patients with ITF who underwent PFNA and found that the total blood loss from the admission day to the first and third postoperative day was 693.5 ± 359.6 mL and 863.8 ± 429.9 mL, of which the corresponding hidden blood loss was 86.8% and 89.4%, respectively.^[7]^ Tian found that 26 of 79 patients who underwent PFNA received 400 mL of blood transfusion and 22 patients received 800 mL of blood transfusion, warning the orthopedic staff to pay attention to perioperative anemia in PFNA patients.^[8]^ Postoperative anemia after fracture tends to cause ischemia and hypoxia in cells, tissues and organs, increasing the rate of blood transfusion, risk of infection, disability and mortality, triggering blood-borne diseases and affecting immune function, delaying postoperative recovery and prolonging hospitalization.^[9,10]^ Blood transfusion is the most effective way of treating anemia, which enhances hemoglobin concentration and improves anemia in a short term, but there are various severe risks such as viral infections, immune allergic reactions, acute hemolytic reactions, etc. Meanwhile, the current clinical blood use guidelines are all restrictive transfusion thresholds (hemoglobin concentration <8 g/dL).^[11]^ Other treatments include nutritional support, iron supplementation, erythropoietin, erythropoietin, and use of hemostatic drugs, and these modalities are prone to various side effects.

Meanwhile, traditional Chinese medicine (TCM) is widely used in the treatment of anemia for activating blood circulation and removing blood stasis. It functions in many ways, such as engaging in multi-linked and multi-targeted regulation, improving the bone marrow hematopoietic microenvironment, restoring bone marrow hematopoietic function, and improving the quality of life of patients. Therefore, this study followed the principles of prospective, randomized, and controlled, aiming to explore the clinical efficacy of TCM methods on postoperative anemia in ITF patients who underwent PFNA, for the purpose of obtaining more completed information on safety and efficacy, and providing research methods and ideas for the treatment of postoperative anemia in ITF.

## 2 Methods

### 2.1 Study registry

**Figure 1:**
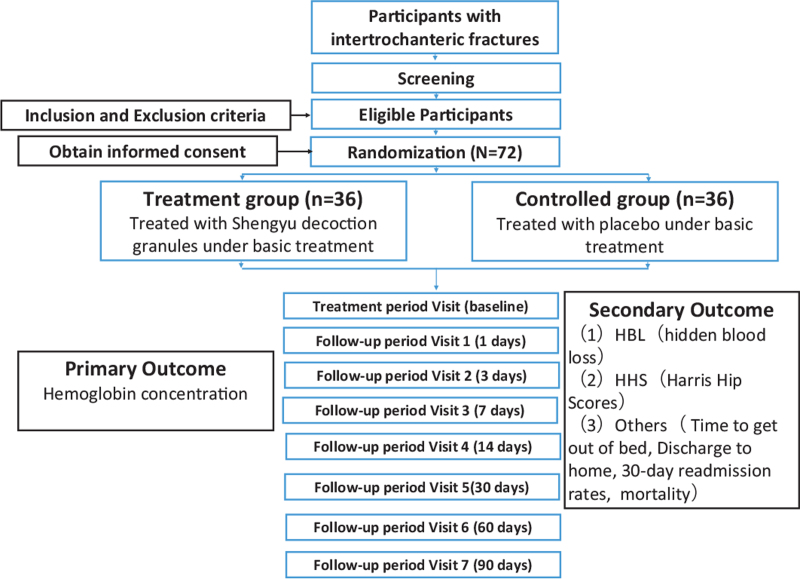
Study Flow.

This research protocol follows the latest consolidated standards of reporting trials 2017 (See Fig. 1 for the flow chart), and Standard Protocol Items: Recommendations for Interventional Trials 2013 statement (see SPIRIT checklist, Supplemental Digital Content Table S1).

### 2.2 Study design

This study is a prospective, outcome assessor-, and data analyst-blinded randomized controlled clinical trial. The objective of this proposed study was to investigate whether Shengyu Decoction could improve the symptoms of anemia after PFNA in elderly ITF patients. Elderly patients with an emergency diagnosis of ITF at our institution from January 2022 to December 2023 were enrolled. All patients were treated with PFNA intramedullary fixation at our hospital.

The principal investigator (PI) is responsible for the overall project and organizing Steering Committee meetings. PIs of sub-center departments are responsible for gathering experts to carry out the project. An independent Steering Committee will be responsible for affairs such as participants’ safety, meetings, recruitment and follow-up of participants, and quality control. The coordinating center is responsible for communicating protocol modifications and providing materials. It consists of a 2-week treatment period and a 6-month follow-up period. Outcome assessment at baseline, post-operative days 1, 3, 7, 14 days, 1 month, 3 months, and 6 months postoperatively. (Fig. 1)

### 2.3 Eligibility criteria

The inclusion criteria are as follows:

1. Patients aged 60 to 85 years.
2. X-ray or CT indicates ITF.
3. Treated with PFNA intramedullary fixation.
4. First day postoperative test results: hemoglobin concentration <8 g/dL.
5. Being willing to give informed consent.^[12]^

The exclusion criteria are as follows:

1. Anemia caused by other diseases.
2. Patients are with open fractures or pathological fracture caused by infection, tumor, or tuberculosis.
3. Patients are with congenital hip dysplasia or osteonecrosis of the femoral head.
4. Failure to understand or sign informed consent.

### 2.4 Intervention

Basic treatments: All patients in both groups were treated with preoperative rehydration, analgesia and anticoagulation, and the relevant departments were consulted to exclude preoperative contraindications; all patients were treated with PFNA intramedullary fixation; postoperative treatments such as rehydration, anticoagulation, analgesia, infection prevention and regular drug changes were given accordingly.

Treatment group: Patients in the trial group were treated with Shengyu decoction granules under basic treatment with 1 dose daily in 2 times for 14 days.

Control group: The control group was treated with placebo on the base treatment, and the placebo was simulated by Shengyu decoction granules, which were required to be free of the active ingredients of the test drug, and their color, taste and odor were similar to those of Shengyu decoction, and 1 dose was given daily in 2 doses for 14 days.

### 2.5 Randomization and allocation

After qualifying for screening, patients will be randomized into 2 groups with an allocation ratio of 1:1. The randomization will be created via SAS PROC PLAN software (SAS Inc., Cary, NC) by an independent third-party clinical research organization (Institute of Basic Research in Clinical Medicine, China Academy of Chinese Medical Science) and hid from the researchers by a senior data manager who is not involved in the study. The group assignment list will be sealed in light-blocking envelopes and be opened by the researchers following informed consent procedures and baseline testing.

### 2.6 Patient population and recruitment procedure

Participants will be recruited from Longhua Hospital affiliated to Shanghai University of Traditional Chinese Medicine. Those patients eligible for enrollment and willing to participate in the study were initially evaluated at baseline and then finally identified through a combination of clinical presentation, physical examination, imaging and laboratory tests. Considering the age of the patients enrolled in this study, the investigator must obtain the consent of the patient's immediate family besides the patient's own informed consent to start the trial. Participants will be informed about the required cooperation in the study, the potential benefits of participating in the study, the possible adverse effects, risks and discomforts of participating in the study, and the associated reimbursement costs and confidentiality of personal information. Participants will also be informed of withdrawal from this study at any time during the study and that the investigator may discontinue your participation in this study at any time out of concern for your best interests. A data collection form including all variables of interest and all potential risks will be completed by the corresponding research center. All information obtained will be kept in an e-database for future statistical analysis. Recruitment will start in January 2022 and is expected to end in December 2023. Final follow-up of all participants will be completed on December 31, 2024.

### 2.7 Ethics

This study is to be conducted in accordance with the principles of the Declaration of Helsinki and has been approved by the Sichuan Regional Ethics Review Committee on Traditional Chinese Medicine. Details of the trial program have been approved by the appropriate Institutional Review Boards. Prior to the clinical trial, the participants will provide written informed consent.

### 2.8 Blinding

All investigators, physicians, nurses, patients, assessors, statisticians, and participants remained blinded to group assignment until the trial ended, when all statistical analyses were finished. If any clinically significant adverse events occur after the first dose that are potentially treatment-related, the study physician will re-evaluate the participant and the PI will determine whether unblinded treatment is necessary. If non-blinding is required, the allocation information will be provided.

### 2.9 Outcome measurements

#### 2.9.1 Primary outcome measurement

Hemoglobin concentration is the main basis for the diagnosis of anemia and visual indicator of the patient's blood volume, therefore we consider it as an essential objective basis for assessing the efficacy of the treatment.

#### 2.9.2 Secondary outcome measurements

HBL: HBL comprises a relatively high percentage of the total perioperative blood loss in ITF patients, which is the dominant cause of postoperative blood loss in intramedullary fixation systems and is strongly associated with postoperative anemia. HBL was calculated based on changes in Hct levels and estimated patient's blood volume (PBV). Following Nadler's formula, PBV (L) = k1∗height (m)^3^ + k2 ∗weight (kg) + k3. For men, k1 = 0.3669, k2 = 0.03219, and k3 = 0.6041, and for women, k1 = 0.3561, k2 = 0.03308, and k3 = 0.1833. In addition, the total red blood cell loss (TRBCL) was calculated according to the Gross formula: TRBCL (L) = PBV∗ (Hctpre − operation − Hctpost − operation), TBL(L) = TRBCL/Hctpre-operation + BVtrans. Finally, it can be derived that HBL(L) = TBL − VBL.^[13–15]^

HHS score: Anemia also interferes with postoperative joint function recovery in hip fracture patients and slows down the overall postoperative rehabilitation progress of patients16.Therefore, the Harris score was used to measure the degree of recovery of the patient's joint function after surgery, indirectly reflecting the patient's anemia. The Harris score consists of 10 questions, with 2 questions in the physician physical examination section (ROM and absence of deformity) and 8 questions in the patient-reported outcomes section.

The other outcomes also included time to get out of bed, discharge to home, 30-day readmission rates, and mortality.

### 2.10 Safety assessments

To ensure the safety of the participants, all data collection was conducted under the supervision of the program director. During each follow-up visit, the patients’ adverse reactions during the drug administration were recorded, especially subjective discomfort reactions such as gastrointestinal, cardiovascular and cerebrovascular reactions, in order to understand whether the drugs existed toxic side effects. In addition, the biochemical indexes of patients, particularly liver function and kidney function indicators, are examined at each follow-up visit to determine whether there is any damage to internal organs from the objective test results.

### 2.11 Sample size calculation

The sample size estimation was based on the mean and standard deviation of the primary efficacy parameters with reference to the pretest. α= 0.025, 1 sided test, and β = 0.10 were established. According to the results calculated by PASS 15.0 software (NCSS, LLC. Kaysville, UT, USA), 30 patients per group were required. Due to an estimated dropout rate of 20%, 36 patients were eventually included in each group.

### 2.12 Statistical analyses

A detailed statistical analysis protocol will be formulated prior to all analyses. All data will be analyzed in the clinical research center of Longhua Hospital affiliated to Shanghai University of TCM by statisticians blinded to allocation using the SPSS 20.0 statistical software (SPSS Inc., Chicago, IL). After evaluating the data distribution, 2-sample t-test (or Wilcoxon rank-sum test) will be used for the analysis of continuous variables. For categorical data, the Chi-Squared test (or Fisher exact test) will be used. Outcome variables including hemoglobin concentration, hidden blood loss, HHS scores for hip joint-specific function will be summarized with means and SDs. For statistical analysis of continuous outcomes, changes in the scores before and after treatments will be regarded as dependent variables, with the baseline scores and research institutions as covariates and the groups as fixed factors, to perform the analysis of covariance. To verify the trends for each visit in the 2 groups, repeated measures analysis of variance will be performed. For categorical outcome data, the Chi-Squared test (or Fisher exact test) will be used.^[16,17]^

### 2.13 Quality control

Prior to the start of the trial, all physicians, nurses, and assessors participating in this study participate in a 1-month training period of 6 hours per day, 5 days per week, for a total of 120 hours. To ensure that all trial participants adequately understand the process of the trial. In addition, a common standard operating procedure (SOP) for the treatment will be provided and practitioners will follow this SOP. At the end of each week, the competency of the participants will be assessed. To guarantee the quality of the whole test, there will be 3 strictly supervised by trained quality supervisors. During the trial, the supervisor reviews case report forms and conducts 2 interventions per month. After verification of the case report form, the data will be input into the database independently by 2 full-time study members.

## 3 Discussion

ITF is commonly occurring in senior citizens, and those who are senior in age generally suffer 1 or more basic diseases, whose nutritional status is already poor. Trauma and surgical stimulation not only aggravate the existing disease or induce corresponding cardiovascular complications, but also worsen the nutritional status, which can easily cause postoperative anemia in patients. Because of the limited clinical modalities available for the treatment of postoperative anemia after fracture surgery, and most of them have various side effects that are not easily tolerated by the elderly. Therefore, from a TCM perspective, we proposed a protocol using mild Chinese herbal decoction to treat postoperative anemia in ITF.

Considering this objective, we conducted a systematic literature search before starting this trial to ensure its comprehensiveness. We have thoroughly searched PubMed, MEDLINE, EMBASE, Cochrane Library, ISI web of knowledge, Wan Fang Data, CNKI databases, Vip Journal Integration Platform (VJIP), and Chinese BioMedical databases from the inception to June 2021.

This trial aims to observe the effectiveness and feasibility of this treatment and explore a way to rapidly correct postoperative anemia in TCM and promote patient recovery. Hopefully, this study will provide a theoretical basis and clinical evidence for the treatment of postoperative anemia after PFNA in elderly ITF patients and explore new ideas and methods for clinical treatment.

## Author contributions

**Conceptualization:** Xuequn Wu.

**Data curation:** Yu Xiao, Yunlu Zhang.

**Formal analysis:** Wei Lu, Yunlu Zhang, Hongbo Wan.

**Funding acquisition:** Yu Xiao.

**Supervision:** Wenhao Zhu, Zhaoxiang Fan.

**Validation:** Wenhao Zhu, Hao Hu, Zhaoxiang Fan.

**Visualization:** Yanqi Feng, Zhaoxiang Fan.

**Writing – original draft:** Hao Hu, Yanqi Feng.

## Supplementary Material

Supplemental Digital Content
